# Falls and risk factors of falls for urban and rural community-dwelling older adults in China

**DOI:** 10.1186/s12877-019-1391-9

**Published:** 2019-12-30

**Authors:** Li Zhang, Zhihong Ding, Liya Qiu, An Li

**Affiliations:** 1 China University of Political Science and Law, Beijing, 102249 China; 2 Central University of Finance and Economics, Beijing, 102206 China; 3 Peking University, Beijing, 100871 China

**Keywords:** Older people, Fall, Risk factors, China, Rural-urban difference

## Abstract

**Background:**

Falls among older people have become a public health concern due to serious health consequences. Despite abundant literature on falls in older people, little is known about the rural-urban differentials in falls among older people in China. This research fills the voids of prior literature by investigating falls and the associated risk factors among Chinese seniors, with a particular focus on the rural-urban differences.

**Methods:**

Data are from the 2010 wave of Chinese Longitudinal Survey on Urban and Rural Elderly. The analysis includes 16,393 respondents aged 65 and over, with 8440 and 7953 of them living in urban and rural areas, respectively. Descriptive analyses are performed to examine incidence, locations, circumstances and consequences of falls in older people. Regression analysis is used to investigate the effects of risk factors on falls among older people in urban and rural China.

**Results:**

The incidence of falls is higher among rural than urban older people. In both settings, older people are more likely to fell outside of home. But common outdoor falls among rural and urban older people differ in terms of locations and circumstances. Urban older people are more likely to report falling on the road whereas their rural counterparts have experienced more falls in the yard. Falls occurring within homes or immediate home surroundings are also common; but few falls occurred in public areas. The rate of hospitalization of urban seniors after falling is higher than that of rural ones. Most risk factors of falls show similar than different effects on rural and urban elders’ risks of falling.

**Conclusions:**

Incidence, locations, circumstances and consequences of falls vary among Chinese rural and urban older people. But most risk factors for falls show similar effects on rural and urban elders’ odds of falling. Implications drawn from this research provide suggestions for the government and local agencies to develop suitable fall prevention strategies which may well be applicable to other countries.

## Background

Falls among older people have become a major public health problem in many countries. According to the World Health Organization’s estimation, there are nearly 424,000 fatal fall incidents each year [1]. It is estimated that approximately 30.0% of people aged 65 years and older have experienced a fall, and about half of them experienced recurrent falls [2]. About one third of community-dwelling people aged 65 and over in the United States experience falls each year, with about 10.0% of falls resulting in serious injuries [3]. Older people who had a fall are also more likely to experience serious complications, resulting in death within the same year 50.0% of the time [4]. Data from the 2014 National Injury Surveillance System (NISS) revealed that in China, for a total of 77,779 accidental injuries among people aged 60 and over, 52.8% of them were caused by falls [5]. Thus far, accidental injuries have become the fourth leading causes of death among Chinese older people; and falls are indeed the major cause of elders’ accidental injuries in China [6]. Senior falls among world countries (including the U.S. and China) have become a serious public health problem [7]. Since the consequences of falls and injuries may result in disability, loss of independence, fear of falling, social isolation, functional decline and mortality [3], exploring patterns of falls among Chinese older people and the associated risk factors is warranted.

According to the 2010 Chinese Census data, by 2010, Chinese population aged 60 and over has reached 178 million, which is about 8.9% of the total Chinese population. Among them, population aged 80 and over was 21 million, which counts 1.57% of the overall Chinese population. Considering such a large population of seniors, it is very important to explore factors that may cause accidental falls that deteriorate older people’s health. A clearer understanding of how personal characteristics, health condition, behavioral and lifestyle factors influence fall rates in Chinese populations is essential for elucidating fall prevention strategies in China. Effective senior fall prevention strategies found in China may well be applicable to other countries, such as Japan, South Korea and the like. The Chinese experience may also be beneficial to some less developed countries as the living condition of rural China resembles that in other developing countries.

Given a huge gap that has long been existing in rural and urban China, investigating the rural-urban differentials in falls among Chinese older people becomes the major concern of this research. The definition of rural and urban residents is based on the household registration status of the respondent. Since the establishment of socialist China, the Chinese government has developed the household registration system to control internal mobility of people moving from the countryside to urban areas. The household registration status is based on the rural or urban residence of citizens. Before the 1980s, residents whose household registrations were in rural areas were not allowed to migrate to cities. The household registration system therefore has served as an “internal passport” to prevent people moving from villages to cities. Such a residential registration system has played an important role in population management and social control, also helped to maintain socioeconomic inequalities. Big gaps exist between rural and urban citizens, with regard to benefits and entitlements. After the 1980s, under the social context of China changed from planned economy to market-oriented economy, have a less restrictive control on rural-to-urban mobility. This is because a tremendous amount of rural labors were demanded by non-government owned sectors in cities. Consequently, a huge number of rural citizens moved to cities. But those migrants often suffered stigmatization and deprivation in various aspects, including job seeking, workplace benefits, and access to medical and other public services. Urban residents in China generally reported higher educational attainments and income, better health, longer life expectancy and more favorable living condition as compared to their rural counterparts [8–12]. Medical resources, including hospitals and medical personnel are readily available in most cities, but not in many areas of the countryside. In addition, although medical insurance in China has expanded rapidly since the turn of the century, reimbursement rates are generally much lower in rural areas [13, 14]. The health disparities and disadvantages in medical insurance for rural residents may have caused different consequences of falls in rural and urban China. This is because unavailability of medical resources in rural areas may have prevented elders from seeking medical treatments immediately after falls. As a result, clinic visits, hospitalization and long-term treatment rates after falls in rural and urban areas differ. The living environments for rural and urban older people are also dissimilar. For example, most rural residents live in more spacious one-story apartments whereas a large proportion of urbanities squeeze in high-rise buildings. Drastically diverse environmental factors could be a reason for dissimilar fall incidence rates among elders residing in cities and the countryside. The cultural tradition, leisure and physical activities among rural and urban seniors are also unalike. Living arrangement patterns in two spheres are divergent as well. There is a higher proportion of urban elders living in institutions or living alone than that of their rural counterparts [15, 16]. Prior research found that having adult children around and being taken care of by adult children reduced Chinese elders’ probability of falling [17]. Thus, the variation in living arrangement patterns could cause diverse senior fall incidence rates in rural and urban China. Considering the rural and urban divide presented in previous literature, this study hypothesizes that the rural-urban gaps discussed above could have resulted in patterns and determinants of falls vary among rural and urban seniors. Before describing patterns of falls among Chinese older people, the paper will first review fall risk factors that have been revealed in previous studies, which provides a guideline for this current analysis.

### An overview of fall risk factors

Falls among older peple have diverse and complex causes. One line of research has grouped fall risk factors among elders into intrinsic and extrinsic categories. The intrinsic risk factors include previous history of falls, cognitive and functional impairment, poor vision, very old age (80 and above), arthritis of knees, poor balance while standing, turning, changing position or walking, use of assistive devices, comorbidities (depression, stroke, Parkinson’s disease, postural hypotension, arthritis), weak hand grip strength, motor weakness (e.g., difficulty in standing up from a chair), gait impairment and medications (the use of hypnotic, anti-depressants or tranquillizers and the use of four or more prescribed drugs) [18–20]. Having disabilities in activities of daily living (ADL) has also been documented to be linked to a higher risk of falling among elders [21]. The extrinsic risk factors are mostly environmental ones that relate to living condition of older people. According to prior analyses, home hazards have been found to be a major factor that resulted in senior falls [22]. Generally, the greater the number of risk factors in an older adult, the higher the risk of falling. It has been reported that the risk of falling increases from approximately 10.0% for those with none or one risk factor to approximately 70.0% for those with four or more risk factors [23].

Another line of research classified fall risk factors into those that have caused indoor and outdoor falls. Studies showed that indoor falls tended to occur among frail individuals, such as women with poor health characteristics. But outdoor falls were more likely to occur among more active people with healthier characteristics, such as fast gait speed and are heavily influenced by outdoor environmental characteristics [24–26]. By studying 2193 individuals aged 45 and over from five Northern California Kaiser Permanente Medical Centers between 1996 and 2001, Li and colleagues found that falls occurred outdoors more often than indoors among most seniors. Physical activity showed a positive association with risks of outdoor falls; and poorer health was revealed to be linked to a greater risk of indoor falls [27]. Kelsey and associates studied 765 women and men, mainly aged 70 and older, from randomly sampled households in the Boston Massachusetts area. They concluded that risk factors for indoor falls included older age, being female, and various indicators of poor health. Risk factors for outdoor falls were younger age, being male, and being relatively physically active and healthy [28].

Although numerous studies have identified factors of falling, few studies have focused on examining whether fall risk factors are similar for rural and urban Chinese older people. Additionally, no study has contrasted incidence, locations, circumstances, and consequences of falls among urban and rural older people in China. To address the above concerns, this study analyzes community-dwelling individuals aged 65 and over drawn from nationally representative data to: 1) reveal the incidence, locations, circumstances, and injuries relating to falls among older people in urban and rural China; 2) contrast environmental and personal risk factors for falls among rural and urban Chinese elders. The study contributes to the literature by improving our understanding on risk factors of falls among Chinese rural and urban elders. The results will be useful for creating optimal fall prevention strategies for older adults in both rural and urban China. Findings from China also provide useful implications for other countries.

## Methods

### Data and sampling strategy

**Fig. 1:**
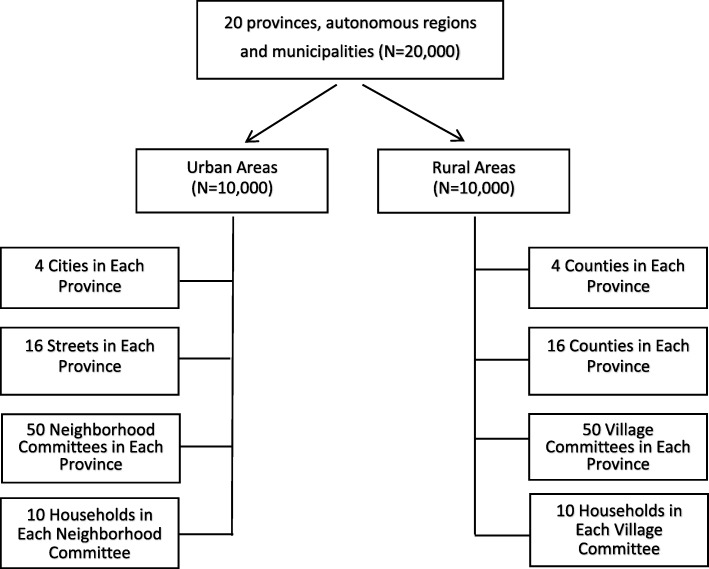
Sampling Scheme of 2010 Wave of the Chinese Longitudinal Survey on Urban and Rural Elderly

Data come from the 2010 wave of the Chinese Longitudinal Survey on Urban and Rural Elderly, conducted by the Chinese Research Center on Aging (CRCA). Since 2000, the CRCA has started surveying community-dwelling rural and urban elders’ personal characteristics and a variety of issues including retirement and employment after retirement, social welfare and security, housing, community-based service programs and utilization, family networks and social participation, medical insurance and health programs, mental health and available psychological consulting services. The 2000 and 2006 waves did not include questions on older people’s fall related information. The 2010 wave is the first one that started collecting information on occurrences, incidence, circumstances, locations as well as consequences of elders’ falls. Based on the distribution of Chinese population aged 60 and over reported by the 2010 Census data, the survey applied multistage proportional random selection strategy to choose respondents from 20 (out of a total number of 31, excluding Hongkong, Macau and Taiwan areas) provinces, autonomous regions and municipalities in China. The 20 provinces, autonomous regions and municipalities include Beijing, Hebei, Shanxi, Liaoning, Heilongjiang, Shanghai, Jiangsu, Zhejiang, Anhui, Fujian, Shandong, Henan, Hubei, Guangdong, Guangxi, Sichuan, Yunnan, Shaanxi and Xinjiang. According to the 2010 Chinese Census data, 49.7% of the population lived in urban areas and the rest of 50.3% lived in rural areas. Thus, the 2010 Wave of the Chinese Longitudinal Survey on Urban and Rural Elderly sampled equivalent numbers of rural and urban elders. In each province, autonomous region and municipality, 500 rural and 500 urban respondents were interviewed. The sampling design is as follows: (1) Randomly choosing 4 cities or 4 counties in each province/autonomous region/municipality (in municipalities, districts are treated as equivalent to cities or counties); (2) In each city or county, randomly selecting 16 streets or villages; (3) In each street or village, randomly choosing 50 neighborhood or village committees; (4) In each neighborhood or village committee, randomly choosing 10 households with at least one senior aged 60 and over to participate the survey interview. Please see Fig. 1 for sampling scheme of the 2010 wave of the Chinese Longitudinal Survey on Urban and Rural Elderly.

The survey was granted ethical approval from Research Ethics Committees of Chinese Research Center on Aging (CRCA). All subjects signed written informed consent before interview. The respondents also agreed that the anonymous information collected by the survey can be released for public use (including publication). Through door-to-door recruitment in randomly sampled households with at least one member aged 60 years or older, the 2010 wave of the survey obtained 20,000 respondents. To be consistent with other countries, this research defined the older population as those aged 65 and over. Accordingly, the analysis includes 16,393 respondents with 8440 and 7953 of them living in urban and rural areas, respectively.

### Variables

In the survey, the respondent was asked if he/she had experienced any falls in the past year. The definition of falls in this research follows the definition given by the World Health Orrganization, that is, “a fall is defined as an event which results in a person coming to rest inadvertently on the ground or floor or other lower level” (https://www.who.int/news-room/fact-sheets/detail/falls). If the respondent answered “yes”, then the frequency of falls was also ascertained. There was also a question asking number of falls in the past year. Reasons, places, and consequences of falls were also asked for the falls during the past 12 months. These questions are used to measure older people’s falls in this research. When using regression models to examine the effects of fall risk factors on elders’ falls, the fall variable is coded as a categorical variable, i.e., whether the respondent reported any falls in the year before the survey year. Coding falls as a categorical rather than a continuous variable is preferred because fall related information was all based on elders’ memories. Thus, it is easier for older people to recall whether they had falls than recalling how many falls they had in the previous year. In this sense, whether they had falls may capture more accurate information than numbers of falls.

The personal characteristics include age, gender, education (1 = illiterate 0 = literate), annual household per capita income, and living arrangements (1 = living alone, 0 = living with others). Marital status is highly correlated with living arrangement patterns of the respondent. To avoid collinearity, marital status measure is not included in the analysis.

Several measures evaluating the respondent’s health status are applied in the study. The first measure is self-rated health, which is derived from the following question: “how would you rate your health status?” The answers are very poor, poor, fair, good and very good. The variable is coded as a continuous variable in the regression analysis.

The second measure is the respondent’s chronic disease status, the questionnaire asked: “how many chronic diseases do you have?” There were 25 types of chronic diseases in the answer list, including hypertension, dyslipidemia, diabetes or high blood sugar, cancer or malignant tumor, chronic lung diseases, liver disease, heart disease, stroke, kidney disease, stomach or other digestive disease, arthritis or rheumatism, asthma et al. In the preliminary analysis, correlation analyses were conducted and the study identified the top 10 chronic diseases that have the highest correlation coefficients with falls. Among the top 10 chronic diseases, the study keeps the top five that have caused the highest fall incidence rates among both rural and urban elders, which are cardiovascular diseases, arthritis, cervical and lumbar spondylosis, cerebrovascular disease, and hypertension.

The third measure of the respondent’s health condition is the vision variable which is coded as a continuous variable (1 = bad, 2 = fair, 3 = good). Cognitive function has been operationalized by a series of questions asking during the past month whether the respondent was not able to: 1) recognize relatives or friends, 2) remember names of relatives or friends, 3) find way home, 4) remember taking keys when going out, 5) remember food is being cooked on the oven. The variable is coded as “1” if the answer was “yes” and “0” if otherwise. The cognitive function variable thus has a minimum score of 0 and a maximum score of 5. Depressive symptoms are assessed by the Geriatric Depression Scale with a score of 8 or above (out of 15) indicating depression. Such a scale has been previously validated in the Chinese older population [29].

The elder’s functioning status is assessed by activity of daily living (ADL) disabilities. It was surveyed by questions: “do you have any difficulty with dressing, bathing, eating, transferring, getting into or out of bed, using the toilet (including getting up and down)?” There are three options for the respondent to choose, which are “no, I don’t have any difficulty”, “have some difficulty” and “cannot do it at all”. The case is coded as 1, 2 and 3, respectively. Then the above items are added as the value of ADL, which ranges from 6 to 18. The higher the ADL disability score, the lower the functioning status.

The environmental factors are measured by three variables. The first variable comes from the question asking whether tap water is available in the household (1 = yes, 0 = no). This variable is considered as a measure of environmental factor because in some rural areas where tap water is not available at home, one needs to get water from the well. If no one helps the elder to get water from well then it is assumed that the likelihood of falling among elders increases. The second variable measures household type of the respondent (1 = high-rise building with escalators, 2 = high-rise building without escalators, 3 = one-story apartment). It is hypothesized that high-rise buildings without escalators increase the odds of older people’s falls as compared to other household types. The last variable measures the respondent’s perceptions towards his/her living conditions (1 = satisfactory, 0 = unsatisfactory). This measure, to a certain extent, captures the living condition of the respondent.

Previous literature indicated that increased physical activity was associated with a decreased risk for chronic conditions (such as obesity and cardiovascular disease) as well as a lower likelihood of falling among older persons [2]. Thus, several physical activity variables are applied in this analysis, investigating whether older people playing Tai Chi, participating in muscle-toning exercises and taking a walk (1 = yes, 0 = no) influence elders’ odds of falling. The respondent’s life style is measured by two variables, that is, whether the respondent had a history of smoking and whether the respondent had a history of drinking (1 = yes, 0 = no). Descriptive information for all variables are presented in Table 1.

**Table 1:** Descriptive Statistics of Variables, R Aged 65 and Over, China

Variable	Urban		Rural	
*Dependent Variable*	%	S.D.	%	S.D.
When R fell in past 12 months**				
Yes	15.0		17.0	
No	85.0		83.0	
*Independent Variables*				
1) Personal Characteristics				
Sex***				
Male	49.2		54.9	
Female	50.8		45.1	
Age***				
65–74	55.4		53.3	
75–84	37.9		38.8	
≥ 85	6.8		7.9	
If illiterate***				
Yes	17.2		46.9	
No	82.8		53.1	
Household income per capita ***	29,051.7	76,549.7	7780.3	20,877.1
If living alone ***				
Yes	17.3		18.3	
No	82.7		81.7	
2) Health Status				
Vision***				
Good	38.5		30.2	
Fair	28.7		29.3	
Bad	32.8		40.5	
Chronic Diseases				
Hypertension***				
Yes	48.2		30.6	
No	51.8		69.4	
Heart Disease***				
Yes	33.4		15.7	
No	66.6		84.3	
Arthritis				
Yes	24.6		24.4	
No	75.4		75.6	
Cervical and lumbar spondylosis***				
Yes	18.4		14.3	
No	81.6		85.4	
Cerebrovascular disease***				
Yes	15.1		9.2	
No	84.9		90.8	
Self-Rated Health***				
Very bad	4.5		7.2	
Bad	16.4		25.0	
Fair	56.3		49.4	
Good	19.6		15.7	
Very good	3.3		2.8	
Cognitive function score (mean)***	1.0	1.4	1.2	1.6
ADL disability damage score (mean)***	6.8	2.1	7.0	2.1
Depressive symptoms ***				
Yes	17.3		34.5	
No	82.7		65.5	
3) Environmental Factors				
If tap water is available ***				
Yes	98.8		65.3	
No	1.2		34.7	
Apartment type***				
One-story apartment	16.4		71.9	
High rise building with elevator or on 1st floor	23.7		14.6	
High rise building without elevator	59.9		13.5	
If satisfied with living condition				
Yes	86.0		86.1	
No	14.0		13.9	
4) Physical Activities				
Taichi***				
Yes	6.2		0.5	
No	93.8		99.5	
Muscle-toning exercises***				
Yes	9.6		1.1	
No	90.4		98.9	
Taking a walk***				
Yes	77.8		62.3	
No	22.2		37.7	
5) Life Styles				
Whether smoked ***				
Yes	31.2		42.7	
No	68.8		57.3	
Whether drank***				
Yes	34.6		41.8	
No	65.4		58.2	
N	8440		7953	

*Source*: 2010 wave of the Chinese Longitudinal Survey on Urban and Rural Elderly

*Note*: R refers to the respondent. * *p* < 0.05; ** *p* < 0.01; *** *p* < 0.001 represent significance levels when conducting Chi-square or T-tests to check rural-urban sample differences

### Statistical analysis

Descriptive analyses are used to show basic information of studied sample. Descriptive results are presented separately for urban and rural subgroups. Statistical tests are also applied to investigate whether the rural-urban differences are statistically significant. Chi-square tests are used for categorical variables and T-tests are applied for continuous variables (see Table 1 for details). When studying incidence, locations, circumstances and consequences of falls among older population, besides contrasting the rural-urban differences, gender differentials are also examined. Since fall related variables are categorical ones, Chi-square tests are applied in this section of the analysis (see Table 2 for details).

**Table 2:** Falls by Residence, Sex and Age Group: R Aged 65 and over, China

Variable	Urban(%)							Rural(%)							
	Sub-Total	Sex			Age Group			Sub-Total	Sex			Age Group			
		Male	Female	*P*-value ①	65–74	75–84	> = 85		Male	Female	P-value ②	65–74	75–84	> = 85	P-value ③
1) Number of falls in past 12 months				<.001							<.001				<.001
None	85.0	87.3	82.9		87.5	82.4	79.2	83.0	84.9	80.6		85.5	81.0	75.4	
Once	7.5	6.6	8.5		7.1	8.0	8.7	6.4	5.6	7.4		6.2	6.6	7.3	
More than once	7.4	6.13|	8.7		5.4	9.6	12.1	10.6	9.5	12.0		8.3	12.4	17.3	
2) Location of falls															
*Outdoor*															
On the road	35.0	37.5	33.1	.10	36.9	36.4	19.5	30.8	34.9	26.9	<.001	33.0	31.4	19.9	<.05
Park	4.2	5.3	3.3	.08	4.8	4.1	1.6	0.5	0.3	0.6	.44	0.7	0.2	0.7	<.001
Stairs	14.9	14.1	15.6	.46	17.9	13.3	8.6	6.9	6.4	7.3	.51	6.9	7.1	6.0	<.001
Yard/community	13.4	13.7	13.2	.78	11.7	14.5	16.4	45.9	42.1	49.6	<.01	43.3	49.2	43.7	<.001
Shopping center	3.8	2.6	4.7	<.05	3.8	4.4	0.8	1.1	1.3	0.9	.54	1.7	0.5	0.7	<.001
*Indoor*															
Bathroom	13.9	14.4	13.6	.65	15.6	15.9	20.3	6.9	7.2	6.6	.67	6.1	7.4	8.0	<.001
Bedroom	23.1	21.2	24.5	.17	15.7	26.2	44.5	21.5	21.3	21.6	.98	16.2	22.4	39.1	.32
Kitchen	5.9	4.6	6.8	.09	5.6	5.7	7.8	9.2	8.4	9.9	.37	9.1	8.3	12.6	<.001
Living room	13.3	13.5	13.2	.85	10.4	13.8	25.0	8.2	7.6	8.7	.49	7.6	8.1	10.6	<.001
Public washroom	2.9	2.7	2.9	<.001	3.0	2.7	3.1	3.3	3.0	3.6	.53	3.2	2.7	6.0	.52
Doorsill	7.6	4.4	10.0	<.001	6.0	9.0	9.4	13.4	11.8	14.9	.10	12.1	14.6	13.9	<.001
*Other*	7.8	11.0	5.5	.08	8.4	7.6	5.5	9.5	11.5	7.5	<.01	12.5	7.4	5.3	.13
3) Circumstance of falls															
Taking a walk	71.0	70.3	71.5	.62	72.9	68.2	74.6	79.1	78.5	79.6	.63	80.0	79.2	74.5	<.001
Getting up and sitting down	16.0	17.0	15.3	.42	11.5	17.4	31.0	12.3	13.0	11.7	.46	9.1	13.9	19.3	<.01
Exercising	6.0	7.3	5.0	.09	5.3	6.6	6.4	5.6	7.9	3.5	<.001	5.5	6.0	4.8	.69
Bathing	4.1	3.3	4.8	.19	3.8	5.0	1.6	2.6	2.3	2.9	.48	1.2	3.3	5.5	<.05
Toileting	13.4	13.3	13.5	.94	9.7	16.9	15.1	12.7	15.3	10.3	<.01	11.5	12.1	20.0	.59
Picking up items	4.8	4.9	4.8	.88	3.8	5.5	6.4	7.0	6.6	7.4	.56	7.2	7.2	5.5	<.05
Housekeeping	8.7	7.3	9.6	.14	9.3	9.0	4.0	10.1	7.5	12.4	<.001	11.3	9.6	6.9	.22
Taking on + off clothes	2.9	2.9	2.9	.99	2.3	3.8	1.6	2.7	2.9	2.4	.60	2.2	2.5	4.8	.69
Other	7.7	8.7	7.0	.11	7.2	8.3	8.0	7.1	7.2	4.8	.20	7.7	4.0	6.9	<.001
4) Consequence of most recent fall				<.05							.66				<.05
No injuries	26.9	30.6	24.2		28.4	27.4	17.1	28.8	30.2	27.4		27.1	30.8	27.6	
Minor injuries	40.8	39.5	41.7		40.5	40.7	42.6	41.2	41.5	41.0		41.5	42.0	37.2	
Clinic visit	15.9	12.9	18.2		16.3	15.4	16.3	16.1	15.0	17.1		18.0	42.0	14.7	
Hospitalization	12.3	13.6	11.4		12.9	11.0	15.5	9.4	9.4	9.3		10.8	8.0	9.0	
Long-term treatment	3.0	2.3	3.5		1.1	4.0	7.8	4.0	3.4	4.6		2.0	4.2	11.5	
Other	1.1	1.1	1.1|		0.7	1.5	0.8	0.5	0.4	0.6		0.5	0.7	0.0	

*Source*: 2010 wave of the Chinese Longitudinal Survey on Urban and Rural Elderly

*Note:* (1) p-value ① represent significance levels when conducting Chi-square or T-tests to check gender differences among urban sample; (2) p-value ② represent significance levels when conducting Chi-square or T-tests to check gender differences among rural sample; (3) *p*-value ③ represent significance levels when conducting Chi-square or T-tests to check gender differences between rural and urban sample. (4) Since multiple falls may have occurred to some respondents, the sum of percentages under “location of falls” / “circumstance of falls” exceeds 100%

The dependent variable, whether the respondent had a fall in the year prior to the survey year, is a bivariate variable, multivariate logistic regression is used to investigate how various factors impact the respondent’s odds of falling. Separate logistic regression models are constructed for rural and urban subgroups. The regression equation is as follows:

$$ \log\;it(p)={\beta}_0+{\beta}_1{x}_1+{\beta}_2{x}_2+\dots .+{\beta}_p{x}_p $$

Where *p* represents the probability that the odds on Y is 1, meaning the respondent has a risk of falling. *β*_0_ is the constant, *β*_*p*_ is the regression coefficient for *x*_*p*_. The regression results are presented in Table 3. The significance level of variables in

**Table 3:** Logistic Regression Results of Fall Risk Factors on Falls: R Aged 65 and Over, China

Variable	Urban			Rural		
	B	S.E.	OR (95% C.I.)	B	S.E.	OR (95% C.I.)
1) Personal Characteristics						
Sex (ref. = female)	−0.29**	0.09	0.75 (0.63, 0.90)	−0.11	0.10	0.89 (0.74, 1.08)
Age	0.02*	0.01	1.02 (1.01, 1.03)	0.00	0.01	1.00 (0.99, 1.02)
If illiterate (ref. = no)	−0.07	0.10	0.93 (0.77, 1.13)	0.02	0.08	1.02 (0.87, 1.20)
Natural log of household income per capita	−0.14***	0.04	0.87 (0.81, 0.93)	−0.04	0.03	0.96 (0.90, 1.02)
Living alone (ref. = no)	0.05	0.09	1.05 (0.88, 1.26)	0.12	0.09	1.13 (0.94, 1.36)
2) Health Status						
Vision	−0.08*	0.04	0.92 (0.83, 0.97)	−0.24***	0.05	0.78 (0.71, 0.86)
Hypertension(ref. = no)	0.13*	0.07	1.14 (1.02, 1.32)	0.09*	0.08	1.09 (1.03, 1.23)
Heart Disease(ref. = no)	0.24**	0.08	1.27 (1.09, 1.47)	0.23*	0.09	1.26 (1.05, 1.51)
Arthritis(ref. = no)	0.29***	0.08	1.33 (1.15, 1.55)	0.14*	0.08	1.15 (1.08, 1.34)
Cervical and lumbar spondylosis(ref. = no)	0.27**	0.08	1.31 (1.11, 1.54)	0.22*	0.10	1.23 (1.01, 1.48)
Cerebrovascular disease(ref. = no)	0.36***	0.09	1.43 (1.20, 1.70)	0.46***	0.11	1.59 (1.28, 1.98)
Self-rated health	−0.30***	0.05	0.74 (0.67, 0.82)	−0.35***	0.05	0.71 (0.64, 0.78)
Cognitive function damage	0.16***	0.02	1.17 (1.12, 1.23)	0.14***	0.02	1.15 (1.11, 1.21)
ADL disability	0.06***	0.02	1.07 (1.03, 1.10)	0.11***	0.02	1.17 (1.08, 1.15)
Depression(ref. = no)	0.19*	0.09	1.21 (1.01, 1.44)	0.16*	0.01	1.17 (1.05, 1.37)
3) Environmental Factors						
Whether tap water is available(ref. = no)	−0.31	0.30	0.73 (0.41, 1.31)	− 0.23**	0.08	0.80 (0.69, 0.93)
Apartment type (ref. = one-story)						
High rise building with elevator or on 1st floor	0.13	0.12	1.14 (0.91, 1.43)	−0.02	0.12	0.98 (0.78, 1.23)
High rise building without elevator	0.15*	0.10	1.17 (1.07, 1.42)	0.33**	0.10	1.40 (1.15, 1.69)
If satisfied with living condition (ref. = no)	−0.31***	0.09	0.73 (0.61, 0.88)	−0.24*	0.10	0.78 (0.65, 0.95)
4) Physical Exercise						
Taichi (ref. = no)	0.10	0.16	1.11 (0.82, 1.50)	−0.12	0.64	0.89 (0.25, 3.08)
Muscle-toning exercises	0.08	0.13	1.09 (0.85, 1.39)	−0.07	0.38	0.93 (0.43, 2.03)
Taking a walk	−0.19*	0.08	0.82 (0.70, 0.97)	0.13	0.08	1.14 (0.98, 1.32)
5) Life Styles						
Whether smoked (ref. = no)	0.11	0.10	1.12 (0.93, 1.35)	0.05	0.10	1.06 (0.87, 1.29)
Whether drank (ref. = no)	0.12*	0.09	1.13 (1.04, 1.36)	−0.02	0.09	0.98 (0.82, 1.18)
cons	−0.63	0.67	0.53 (0.14, 1.98)	−0.69	0.57	0.50 (0.16, 1.54)
N	7133			6105		
Chi - square	536.30			567.11		
Log likelihood	- 2778.17			- 2536.03		
Nagelkerke R Square	0.08			0.10		

*Note*:* *p* < 0.05; ** *p* < 0.01; *** *p* < 0.001. Source: 2010 wave of the Chinese Longitudinal Survey on Urban and Rural Elderly

logistic regression models is as follows: * *p* < 0.05, ** *p* < 0.01, *** *p* < 0.001. Stata 14.0 is the statistical software used to conduct the analysis.

The final step of the analysis is to explore whether regression coefficients for the urban subgroup are significantly different from those for the rural subgroup. The research uses Z-tests to perform the analysis. Z value is calculated by the following formula:

$$ Z=\frac{b_1-{b}_2}{\sqrt{SE{b_1}^2+ SE{b_2}^2}} $$

where *b*1 is the regression coefficient of independent variable X for group 1 (urban subgroup), *b*2 is the regression coefficient of the same variable X for group 2 (rural subgroup), and SEb1 and SEb2 are the coefficient variances associated with the first and second groups, respectively. The calculated Z test values for the fall risk variables in logistic regression models are presented in Table 4. If the absolute value of Z for any one variable is less than 1.96, this indicates that the null hypothesis is accepted, i.e., the coefficient in one regression model is the same as the coefficient in another model. If the Z test value is greater than 1.96, the null hypothesis is rejected, signifying that the coefficient in the equation predicting the dependent variable is significantly different from the coefficient in the equation predicting the other. A rejection of the null hypothesis for a particular independent variable that its coefficients are the same in two regression models is also indicated by “No.” An acceptance of the null hypothesis that the coefficients are the same is indicated by “Yes.”

**Table 4:** Z-Tests to Determine if Regression Coefficients are Significantly Different for Urban and Rural Subgroups: R Aged 65 and Over, China

Variables	Z Value	*b*_*1*_ *= b*_*2*_ (Coefs are the same)
1) Personal Characteristics		
Sex (ref. = female)	−1.34	Yes
Age	1.41	Yes
If illiterate (ref. = no)	−0.70	Yes
Natural log of household income per capita	−2.00	No
Living alone (ref. = no)	−0.55	Yes
2) Health Status		
Vision	2.50	No
Hypertension(ref. = no)	0.38	Yes
Heart Disease(ref. = no)	0.08	Yes
Arthritis(ref. = no)	1.33	Yes
Cervical and lumbar spondylosis(ref. = no)	0.39	Yes
Cerebrovascular disease(ref. = no)	−0.70	Yes
Self-rated health	0.71	Yes
Cognitive function damage	0.71	Yes
ADL disability	−1.77	Yes
Depression(ref. = no)	0.33	Yes
3) Environmental Factors		
Whether tap water is available(ref. = no)	−0.26	Yes
Apartment type (ref. = one-story)		
High rise building with elevator or on 1st floor	0.88	Yes
High rise building without elevator	−1.27	Yes
If satisfied with living condition (ref. = no)	−0.52	Yes
4) Physical Exercise		
Taichi (ref. = no)	0.33	Yes
Muscle-toning exercises	0.37	Yes
Taking a walk	−2.83	No
5) Life Styles		
Whether smoked (ref. = no)	0.42	Yes
Whether drank (ref. = no)	1.10	Yes

*Note*: H_0_: *b*_*1*_ for urban subgroup = *b*_*2*_ for rural subgroup; yes = coefficients are the same

## Results

### Descriptive results

#### Incidence of falls

**Fig. 2:**
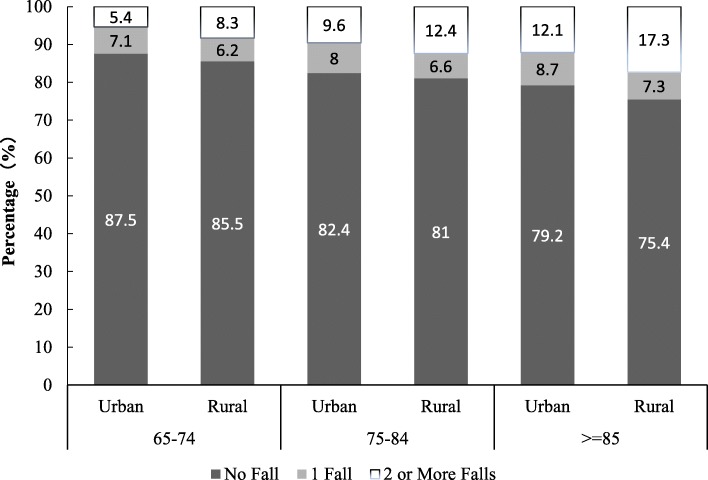
Percentage of Falls by Age Group and Residence: China

As Table 2 shows, at least one fall occurred in previous 12 months among 14.0 and 17.0% of urban and rural respondents, respectively. Overall, rural elders reported a slightly higher percentage of falls. The percentage of women who fell is higher than that of men among both rural and urban respondents. Figure 2 indicates that the proportion of falls increases with age among both rural and urban subgroups. In each age group, the percentage of fallers is higher among rural than urban groups. For instance, the proportion of urban elders aged 65 to 74 who fell more than once is 5.4% and the correspondence rate is 8.3% among rural elders. Another important finding is that with age increasing, the percentages of respondents who fell more than once increase drastically. For example, urban multiple fallers aged 65 to 74 count about 5.4% of all respondents, such a percentage almost doubles for age group 75 to 84, and it amounts to 12.1% among urban subgroup aged 85 and over. The findings suggest that incidence of falls increases with age. The age effect on the rate of falls is particularly strong among multiple fallers. Chi-square tests show that differences in fall incidence between the rural and urban subgroups and between males and females are statistically significant. Thus, one has 95% confidence to claim that for the studied Chinese sample, urban elders are less likely to fell than their rural counterparts; and males are less likely to fell than females in both rural and urban areas.

#### Locations of falls

Since multiple falls may have occurred to some respondents, the sums of percentages under “location of falls” and “circumstance of falls” exceed 100%. Table 2 shows that the percentage of falls on the road is the highest among all locations (35.0% of urban elders and 30.8% of rural elders fell on the road, respectively), followed by stairs, yard/community, and bedroom and living room within their homes or immediate home surroundings. A large proportion of falls occurred on surface levels such as bedroom, living room and kitchen. Relatively few falls occurred in public restrooms/shower rooms, shopping centers and parks.

**Fig. 3:**
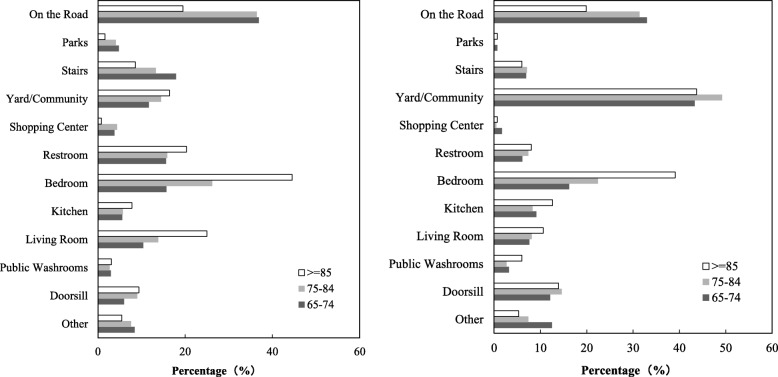
**a** Location of Falls: Urban Seniors, China. **b** Location of Falls: Rural Seniors, China

Results show that the location of falls is related to age, sex and residence. The number of falls occurring outside of home decreases with age. A corresponding increase occurs in the number of falls occurring inside of home. In general, more women fell inside of home than men. These findings are consistent with findings of previous research [30, 31]. The results indicate that the occurrence of falls is associated to exposure. Falls are likely to occur in situations where older people are undertaking their daily activities. According to Figs. 3-1 & 2, the most noticeable rural-urban difference is falls occurring in yard/community (45.9% of rural elders fell in yard/community vs. 13.4% of urban elders fell in yard/community). This is probably caused by different household structures in rural and urban China. Rural dwellers often have spacious yards whereas most urban dweller don’t, which may have resulted in the proportion of falls in yards/communities being much lower among urban than rural respondents. The proportions of elders who fell in parks, shopping centers, restrooms, living rooms, and on stairs are higher among urban than among rural seniors. Such differences could be caused by divergent life styles among urban and rural residents. Chi-square tests indicate that the differentials shown in most locations of falls between the rural and urban subgroups are statistically significant. The differences shown in falls that occurred on the road and in the yard/communities only demonstrate significant differences between male and female elders in rural areas. This means that rural males have a higher risk of falling on the road whereas rural females have higher odds of falling in the yard/community. Significant gender differences also exist in terms of urban elders’ falls that occurred in public washroom and doorsill. The likelihood of falling in public washroom and doorsill for urban female elders is higher than that for their male counterparts.

#### Circumstances of falls

Respondents aged 65 and over have suffered most falls when taking a walk. “Fell while walking” constitutes over 70.0% of the reasons for falls (71.0% vs. 79.1% among urban and rural respondents, respectively). Sitting-down and getting-up, going to toilet, house-keeping and exercising also tend to be common circumstances of falls. The Chi-square tests reveal that circumstances of falls among older people show significant rural-urban differences. To illustrate, urban elders are more likely to fell when getting up and sitting down, bathing, and doing other activities. Whereas rural elders have higher odds of falling when taking a walk and picking up items.

**Fig. 4:**
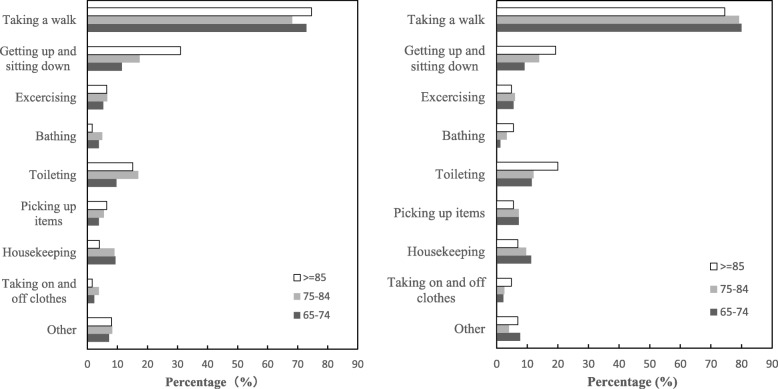
**a** Circumstance of Falls: Urban Seniors, China. **b** Circumstance of Falls: Rural Seniors, China

The circumstances of falls also vary by sex. More women than men fell when they were doing house-keeping related activities. More men than women fell when they were exercising, getting-up and sitting down. But Chi-square tests prove that the gender differentials are only shown among rural elders. Rural older men have a higher likelihood of falling when toileting; but rural older women show a higher risk of falling when picking up items. The circumstances of falls vary by age as well. Figures 4-1 and 2 indicate that with age increasing, the percentages of elders aged 85 and over who fell when getting up and sitting down as well as toileting become higher than other age groups. These findings suggest that diverse prevention strategies targeting towards different sexes and age groups are warranted.

#### Consequences of falls

Falls may cause serious consequences. The results show that 12.3% of urban and 9.4% of rural older people reported hospital admission after the most recent falls. About 3.0% of urban and 4.0% of rural older people experienced long-term care after falls. The percentages of elders who needed clinical visits are 15.9 and 16.1%, respectively. About two-fifth of older people (40.8% of urban respondents and 41.2% of rural elders) reported minor injuries. The rest of the sample reported no injuries (26.9% of urban elders vs. 28.8% of rural elders). Readily available hospitals and medical resources in cities than in countryside may be a reason that has caused a higher hospitalization rate among urban elders. But based on Chi-square tests, no significant rural-urban differences are found in terms of consequences of falls. Significant gender differences are only found among the urban subgroup.

#### Risk factors of falls

Now the paper turns to the analysis of risk factors of falls. The article first presents the descriptive results of fall risk factors. The risk factors are grouped under five categories, which are personal characteristics, health status, environmental factors, physical activities and life styles. Table 1 shows that among the respondents, a slightly higher percentage of men are included among the rural subgroup. About half of the respondents are aged 65 to 74; and the oldest-old group is only 6.8 and 7.9% among the urban and rural subgroups, respectively. Nearly half of the rural respondents are illiterate whereas only 17.2% of the urban samples are illiterate. The household per capita income is much higher in urban than in rural areas. About 83.0% of the respondents reported living with others and the rest of them either lived alone or were in institutions. Chi-square tests and T-tests results indicate that significant rural-urban differences exist in all of the above personal characteristics varaibles.

As to the respondents’ health status, urban seniors reported a better vision than their rural counterparts. As compared to rural elders, higher percentages of urban elders reported having hypertension, heart disease, cervical and lumbar spondylosis and cerebrovascular disease. Urban respondents show a better average cognitive score than rural ones. The mean ADL disability score is higher for rural than for urban respondents (7.0 vs. 6.8). A higher proportion of rural respondents experienced depressive symptoms than their urban counterparts (34.5% vs. 17.3%). Chi-square test and T-test results indicate that except for the arthritis variable, significant rural-urban differences exist in all of the above health status varaibles. Overall, urban older people’s health status is better than that of their rural counterparts.

The environmental factors show significant rural-urban disparities. Nearly one third of rural elders claimed that tap water was not available in their households whereas only 2.0% of urban families had no access to tap water. The apartment types also vary drastically in urban and urban areas, with a majority of rural elders living in one-story apartments and urban dwellers living in high-rise buildings. Chi-square tests display that significant rural-urban differences exist in two of the above environmental varaibles (apartment type and availability of tap water). More urban elders reported having physical exercises than their rural counterparts. The percentages of smokers and drinkers are both higher among the rural subgroup. Chi-square tests show that significant rural-urban differences exist in all physical activity and life style dimensions studied in the research.

### Regression results

Table 3 presents logistic regression results of fall risk factors on falls among rural and urban senior subgroups. With regard to personal characteristics, the results show that among the urban subgroup, men are 25.0% less likely than women to fall. With every 1 year increase in age, the odds of falling increase by 2.0%. For seniors who reported higher income, their risks of falling are also significantly reduced. Education or living arrangement patterns (whether living alone) does not have a significant effect on falls. None of the personal characteristics are found to be associated with older people’s falls among the rural subgroup.

All health status indicators show significant influence on both rural and urban elders’ risks of falling. With the vision score increasing by every one unit, the risks of falling decrease by 8.0 and 22.0% among urban and rural elders, respectively. Similarly, with every one level increase in the respondent’s self-rated health, the risks of falling among urban and rural elders drop by 26.0 and 29.0%, respectively. Both cognitive and ADL impairment are significantly associated with a higher risk of falls. Elders with depressive symptoms are 21.0 and 17.0% more likely to experience falls as compared to those without such symptoms among urban and rural elders, respectively. The presence of chronic diseases, including hypertension, cardiovascular disease, arthritis, and lumbar vertebrae disease are associated with an increased risk of falls among both rural and urban respondents.

The odds of falling decreases 20.0% for those who reported having tap water available at home. This is because unavailability of tap water may force older people to go out and get water from nearby wells. This activity increases the likelihood of falling, especially for those seniors who live alone. As compared to the respondents who lived in one-story apartments, those who lived in high-rise buildings without escalators tend to have about 1.2 and 1.4 times of risks of falling in urban and rural areas, respectively. Fallers tend to be unsatisfied with their living conditions as compared to nonfallers. This is perhaps because unsatisfactory living condition usually links to some uncomfortable features, such as having no access to tap water, lacking of escalators in older apartments et al. These factors could cause elderly falls. Taking a walk, a variable measuring the respondent’s physical activities, is found to decrease the risk of falls by about 18.0% among urban elders. No association was observed between practicing Tai Chi and aerobics and the occurrence of falls. Smokers do not show a higher risk of falls. But drinkers are 1.1 times more likely to fall than non-drinkers among the urban subgroup. These results suggest that most risk factors of falls based on studying Western elders are applicable to urban older people based on studying the sample. But only health status and environmental measures show significant effects on rural elders’ falls.

The Z-values in Table 4 indicate that for the most part the fall risk factor variables’ effects on urban older people’s odds of falls are not different from their effects on rural elders’ risks of falling. Among the personal characteristics variables, only income plays a more important role predicting odds of urban elders’ falling. And among the health status variables, vision has a greater effect on rural elders’ risks of falling. Of the three physical exercise variables, only the walking variable shows a much stronger impact on urban elders’ risks of falling as compared to its effect on rural elders’ fall risks. This suggests that in China, despite higher incidence of falls among rural than among urban older people and the significant rural-urban differences for almost all fall risk factors, the studied fall risk variables account for similar amount of variation in rural and urban eldrs’ odds of falling. And the effects of the independent variables predicting urban and rural elders’ risks of falling are more similar than different.

## Discussion

This research has studied incidence, locations, circumstances and consequences of falls and has further contrasted risk factors associated with falls among Chinese community-dwelling rural and urban elders aged 65 and over. Findings suggest that more falls occurred among rural than urban seniors; and the fall incidence rate is higher among females than males. In addition, more falls occurred outside than inside of the home environment, with the percentages of elders falling on the road (35.0%) and in the yard (45.9%) being the highest among urban and rural elders, respectively. Meanwhile, about 71.0% of the urban respondents and 79.0% of the rural respondents reported “walking” as the circumstances for falling. The regression results indicate that all environmental measures show significant effects on the odds of falling among both rural and urban subgroups, emphasizing an important role of environmental factors predicting senior falls. This finding somehow differs from Svensson, Rundgren and Landahl’s (1992) study which showed a high proportion of falls occurring indoors [32]. The discrepancy can be caused by a relatively younger age structure of our sample. Indeed, recent studies on Chinese elders’ falls and risk factors by Pi and associates (2015) and Wang et al. (2019) echoed the conclusion of this study [33, 34]. Through analyzing sample collected from surveying residents in 68 assisted living facilities in Houston, Chicago and Seattle in the United States, Lee and colleagues (2019) further revealed that adequately designed walkways and higher comfort levels when using outdoor areas were helpful in reducing elders’ fear of falling, which can effectively reduce the risk of senior falls among assisted living residents [35]. Given findings of our research and other studies, enhancing environmental conditions, including better design and maintenance of sidewalks, walkways, streets, parks and recreational places is highly recommended. Regression analysis of this research has shown that availability of tap water and elevator in high rise buildings can significantly reduce the odds of falling among elders. Thus, it is essential to advance basic construction of infrastructural facilities. The fall prevention strategies drawn from this research fall in line with some fall prevention schemes proposed by the World Health Organization (WHO) [36]. Fall prevention strategies drawn from this research may be applicable to other societies.

As to falls occurred in the home environment, some of them were under the circumstances that can be potentially modifiable as well. Home harzard assessment and modification are also suggested. Prevention may focus on risks for falling that arise when seniors are performing regular activities even though these may be perceived as low-risk events. These activities are such as dressing, cooking, toileting, transferring at home et al. Developing public health education programs targeting Chinese elders to increase their fall safety awareness and knowledge is merited.

Besides environmental factors, the research also highlights the role of personal characteristics and life styles in determining elders’ falls. The results show that better health, exercising and higher socioeconomic status (SES) can significantly reduce the odds of falling among Chinese elders. These findings imply that encouraging physical exercises and healthier life styles should be part of the education and self-management programs that prevent older people’s falls. The World Health Organization (WHO) also had similar suggestions in its fall prevention reports [36].

The main part of this research has concerned whether factors associated with senior falls are different between rural and urban residents. Thus, the study applied factors that have been found influential on Western elders’ risks of falling to predict Chinese rural and urban elders’ odds of falling. Findings show that all five dimensions of fall risk factors are significantly associated with urban elders’ falls. Except for income, vision and walking variables, all other fall risk factors show similar effects on urban and rural elders’ risks of falling. In general, better physical and mental health as well as a higher level of satisfaction towards living environment lead to a lower risk of falling. The paper concludes that although rural and urban Chinese elders have significantly different incidence, locations, circumstances and consequences of falls, most risk factors of falls do not differ significantly. In this sense, senior fall prevention strategies that are found effective and successful among urban seniors should be applicable to rural Chinese elders and vice versa.

Although gender diffrences in elderly falls is not a major concern of this research, the analysis does show some gender differentials in terms of locations, circumstances and consequences of falls. The finding reminds us that when the government and organizations are building programs to prevent senior falls, gender differences should not be ignored. We acknowledge that significant social changes have occurred since 2010 when the survey data were collected. For instance, the percentage of elder population aged 60 and over changed from about 8.9% (178 million) of the total Chinese population in 2010 to 17.3% (241 million) by the end of 2017. Aging related issues become even more urgent today. Meanwhile, the Report released by Chinese Aging Research Center pointed out that along with the improvement of medical treatment in the past 10 or so years, medical insurance coverage has been expanding, especially in rural areas. Medical expenditure has largely increased [37]. Improvement in medical treatment may have possibly lowered death rates caused by falls among Chinese elders. The third major social change among seniors is that under a fast trend of urbanization in China, the urban-rural elder population has been redistributed, with the percentage of urban elders increasing among the total senior population. This transition is associated with the change that the overall living condition of Chinese citizens has improved in both urban and rural settings. Tap water has become available in more rural families. These changes may have played a positive role in reducing elder falls, especially falls occurred to rural seniors. Despite the above improvement in the past 10 or so years, the Report by Chinese Aging Research Center also indicated some negative sides. The most apparent one is that the life style of Chinese elders has become more sedentary, with 50% of them reporting never excercising [37]. Meanwhile, more elders enjoy surfing the internet, doing online shopping, chatting on the website and et al. Since this research has discussed how environmental factors and life styles may influence elders’ risks of falling, findings of this research have important implications for elderly fall prevention because social changes discussed above related to the environmental and life style dimensions.

This study has several limitations. First, the data are based on a cross-sectional survey. As other cross-sectional studies, there is an inherent weakness of not being able to separate the cause-effect relationships of variables. In addition, recall bias may exist, especially among those elders who have poor memory. Since the data were obtained on the basis of past-year recall and only the most recent fall was queried in detail, it is possible that the frequency of falls was underreported. Prior research has shown that past-year falls are likely to be underreported by 13.0 to 32.0% [38]. Some errors may have occurred when reporting the locations, incidence and circumstances of falls. Thus, findings of this research need to be replicated by other researchers. A prospective study is warranted to identify the incidence, risk factors, and circumstances of falls. Since the main focus of the survey was not on falls of older people, the questionnaire was not able to exhaust all potential fall risk factors, such as medications the elders took, balance while standing, turning, changing position or walking, use of assistive devices et al. Future research can further examine cultural factors on falls, for example, the effect of squatting on fall incidence. The measures of environmental factors are also scarce. These have prevented the current study from exploring fall risk factors in a more comprehensive manner. Even with the limitations, this study is one of the first ones that analyze nationally representative data to identify occurrence, locations, circumstances, consequences and risk factors of falls among Chinese community-dwelling elders in both rural and urban areas. Findings of this research offer an important base for conducting future prospective studies and launching possible senior fall prevention programs in China and possibly other countries. The government and local agencies may consider applying some public health approaches to falls prevention. For example, reduction of income inequality, allocating more resources to disadvantaged groups et al. These factors are outside traditional individual centred clinical approach to falls prevention but they should still be considered.

## Conclusions

This is one of the first studies that contrast incidence, locations, circumstances and consequences of falls among rural and urban Chinese community-dwelling elders aged 65 and over. The study focuses on exploring whether fall risk factors differ among rural and urban elders in China. Results have revealed that incidence, locations, circumstances and consequences of falls vary among Chinese rural and urban older people. But most risk factors of falls show similar effects on rural and urban elders’ odds of falling. The findings imply that successful fall prevention strategies based on urban experience in China can also be utilized to decrease falls among rural older people and vice versa. This research will be useful for creating optimal fall prevention strategies for older adults in China and maybe other countries as well.

## Data Availability

This article is based on a publicly available dataset derived from the 2010 wave of the Chinese Longitudinal Survey on Urban and Rural Elderly, conducted by the Chinese Research Center on Aging (CRCA). The dataset can be obtained after sending a data user agreement to the data team.

## Abbreviation

ADL: Activities of daily living.
